# Longitudinal Use of the Consolidated Framework for Implementation Research to Evaluate the Creation of a Rural Center of Excellence in Transgender Health

**DOI:** 10.3390/ijerph17239047

**Published:** 2020-12-04

**Authors:** Pamela J. Tinc, Christopher Wolf-Gould, Carolyn Wolf-Gould, Anne Gadomski

**Affiliations:** 1 Bassett Healthcare Network, Research Institute, 1 Atwell Road, Cooperstown, NY 13326, USA; anne.gadomski@bassett.org; 2 Bassett Healthcare Network, Susquehanna Family Practice/Gender Wellness Center, 1 Atwell Road, Cooperstown, NY 13326, USA; christopher.wolf-gould@bassett.org (C.W.-G.); carolyn.wolf-gould@bassett.org (C.W.-G.)

**Keywords:** transgender health, rural health, center of excellence, implementation, organizational culture, Consolidated Framework for Implementation Research

## Abstract

Background: Transgender people face numerous barriers to accessing care, particularly in rural settings. Transportation, travel time, a lack of providers offering transgender care, and discrimination all contribute to these barriers. The Gender Wellness Center was established in New York State, USA, to fill a gap in rural transgender care and was subsequently awarded a Robert Wood Johnson Foundation grant to establish a Center of Excellence. This study examined the implementation of the Center of Excellence, a complex intervention, to assess barriers and facilitators to implementation over 18 months. Methods: The Consolidated Framework for Implementation Research (CFIR) was used to develop baseline and follow-up surveys. These were distributed to members of the core implementation team at the Gender Wellness Center at the midpoint and conclusion of the Robert Wood Johnson Foundation grant. Responses were largely open-ended and analyzed qualitatively. Results: Results are presented in terms of CFIR domains and constructs, as well as the relative outlook (positive or negative) of implementation. Overall, there were improvements over time, with more encouraging feedback and examples of success at follow-up. Though true, organizational culture and individual beliefs about the provision of transgender care challenged implementation of the Center of Excellence throughout the project. Conclusions: This study highlights the importance of organizational culture on implementation efforts, as well as the need for complex, multifaceted interventions to overcome such challenges in order to improve care for marginalized populations.

## Contributions to the Literature

This study highlights the longitudinal use of the Consolidated Framework for Implementation Research for evaluation purposes.

This study explored the influence of organizational culture on implementation of stigmatized interventions in rural settings.

This study describes the benefits and challenges of implementation efforts led exclusively by clinical staff members.

Trial registration: Not applicable.

## 1. Background

Rural settings present numerous challenges around healthcare access for all patients, including long travel times, lack of specialized clinicians, lack of public transportation, and limited capacity for patient care [1]. Transgender patients face particular disadvantages while attempting to access care in rural settings [2]. Rural settings often lack specialty clinics and trained clinicians for transgender patients. Instead, care is provided by clinicians who are unfamiliar with transgender people and their specific healthcare needs [3,4,5]. Transgender patients may avoid seeking medical care due to a history of traumatizing interactions with healthcare providers [6] and the inability to locate affirming providers. Transgender people also report abuse and discrimination from healthcare workers in response to their nontraditional gender identities and sexual preferences [6,7,8]. The development of healthcare systems serving transgender people is considered a “wicked problem”, i.e., one that is systematic, complex, and persistent [9]. As such, multifaceted and complex interventions such as the one described in this study are needed to improve care for transgender people, particularly in rural settings [9].

The Gender Wellness Center (GWC), located in rural upstate New York, was first established in 2015 and is a program of Susquehanna Family Practice. This primary care practice operates within a large multidisciplinary outpatient facility, the Fox Care Center, which is owned and operated by the eight-county Bassett Healthcare Network [10]. These entities provide not-for-profit healthcare services for all patients. Activities within the GWC are led by a clinical director and mental health director and guided by input from a community advisory board. This setting is unique in that other centers for transgender health are typically found within a specialty care setting.

The establishment of the GWC was based on a growing community of transgender individuals seeking care from this practice, which is located nearly 200 miles northwest of New York City in a rural community that is devoid of other transgender-specific health services and where transgender service organizations are limited compared to large urban areas. One doctor provided care to one transgender patient in a family practice in 2007, an event that eventually led to the creation of the GWC as an embedded entity within that family practice. Over time, as additional patients presented asking for services, the clinician began to work with a local mental health provider to offer interdisciplinary care.

The clinicians became acutely aware of the unmet needs of this growing patient population, one with significant barriers to care and healthcare disparities. They recruited additional clinicians and searched for funding. In 2015, they were awarded a Robert Wood Johnson Foundation (RWJF) Clinical Scholars grant, with the goal of establishing a Center of Excellence (COE) in Transgender Health, the first of its kind in a rural area of the US [11].

**Figure 1:**
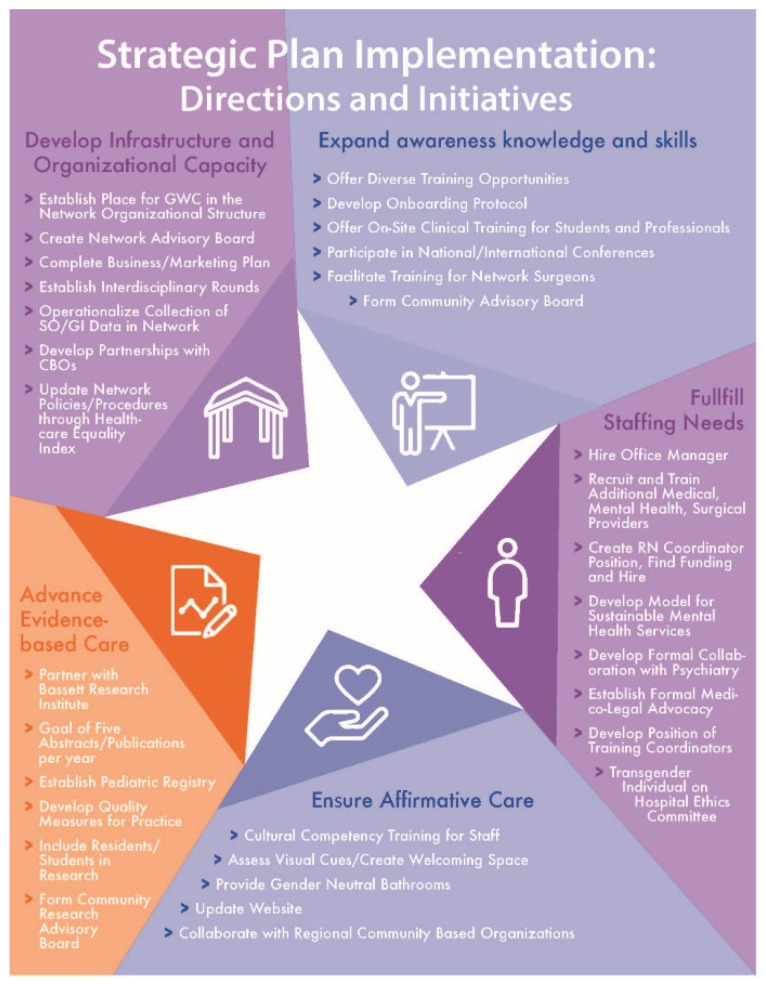
The five COE implementation focus areas and strategic plans for each. Figure originally published in “Leading Community Based Changes in the Culture of Health in the US: Experiences in Developing the Team and Impacting the Community (9)”.

**Figure 2:**
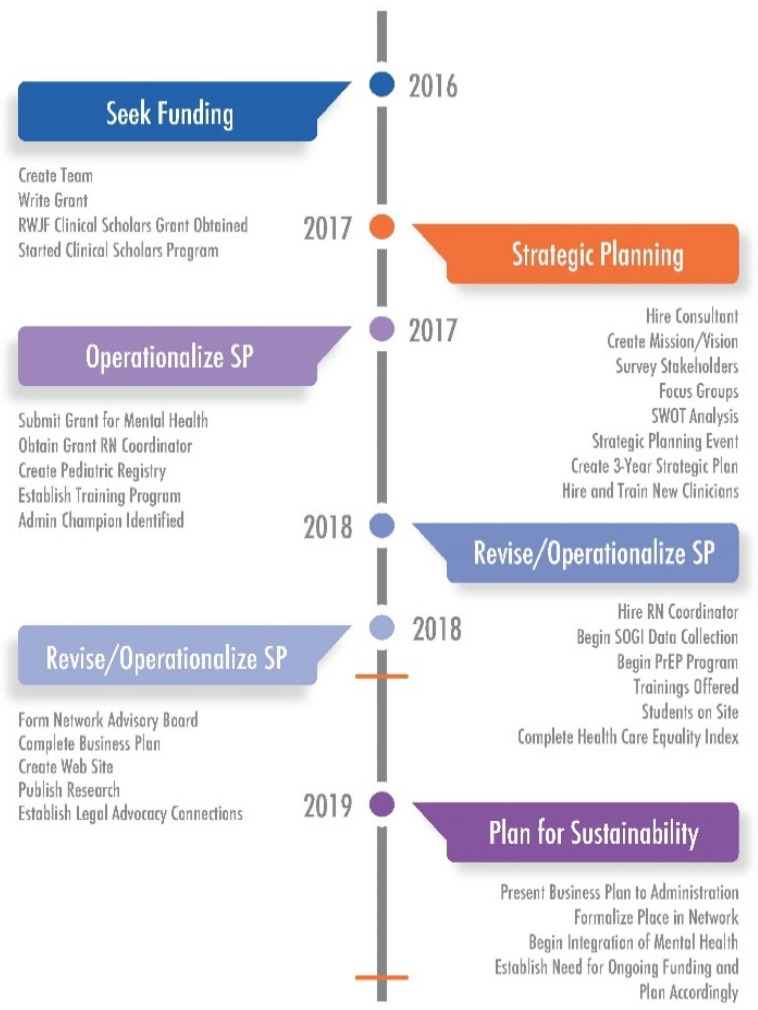
Timeline for implementing the COE. Figure originally published in “Leading Community Based Changes in the Culture of Health in the US: Experiences in Developing the Team and Impacting the Community (9).” Horizontal, orange lines represent the timing of baseline (2018) and follow-up (2019) surveys.

The goal of COEs is to develop specialized skills in an area of practice in order to improve outcomes for patients [12]. To develop this COE, the GWC team first developed a mission and vision statement and identified six essential objectives, or prongs, for their center including the provision of trans-specific (1) medical care, (2) surgical care, (3) mental healthcare, (4) community-based research, (5) legal services, and (6) training and education. The team then participated in a three-part strategic planning retreat led by an external facilitator. The facilitator was a professional strategic planning consultant with a Master’s of Social Work degree. The strategic planning process included three 1–2-day, off-site retreats attended by three GWC medical providers, a case worker, three mental health providers, and the professional consultant. The initial session focused on brainstorming steps required for development and implementation of the Center of Excellence. Five strategic initiatives were agreed upon. Specific tasks related to these initiatives were identified and assigned to team members. Follow-up retreats, supplemented by monthly team meetings, were scheduled to monitor task completion. Figure 1 highlights the five arms of the strategic plan and key activities within each, while Figure 2 shows the grant timeline, including two time points relevant to this study.

Efforts such as this one that address wicked socio-cultural problems with numerous moving parts are difficult to evaluate; however, in order to improve implementation efforts related to both the current effort as well as future initiatives, evaluation is necessary. The Consolidated Framework for Implementation Research (CFIR) evaluates implementation using several perspectives to explain what works, what does not work, and why in various settings [13]. The CFIR is comprehensive in that it includes a range of implementation factors compiled from various other implementation frameworks and is, thus, suitable for the evaluation of complex initiatives such as the implementation of a COE in rural transgender care [13]. Because of its comprehensive nature, the CFIR provides a framework to evaluate a multitude of potential barriers and facilitators (organized as constructs) to implementation efforts, including in healthcare settings [14,15]. CFIR has been described as “a powerful longitudinal evaluation tool that facilitated capture of thoughtful nuances and key voices throughout the implementation process [16].”

### Study Aims

The aims of this study were (1) to evaluate the implementation of the COE using the CFIR at two points in time during the grant cycle, i.e., at the midpoint and at the end of the grant (Figure 2) and (2) to understand changes in barriers and facilitators to COE implementation over 18 months within the context of rural transgender healthcare.

## 2. Methods

### 2.1. Study Participants

Participants in this study included the eight GWC team members, who dually served as the core implementation team for the COE. These individuals included four medical providers, three mental health providers, and a nurse coordinator. Because the study focused on specific details of the implementation process, those who were not integrally involved in implementation of the COE, i.e., those working within the healthcare network but outside of the GWC, were excluded from the study.

### 2.2. Survey Development

The survey instrument was developed using validated measures of Consolidated Framework for Implementation Research (CFIR) constructs provided at www.CFIRguide.org. Briefly, the CFIR describes implementation settings in terms of five domains: characteristics of individuals (involved in implementation), inner setting (e.g., people, organizations, and processes that directly influence implementation), outer setting (e.g., people, organizations, and processes that indirectly influence implementation), intervention (COE) characteristics, and (implementation) process. Each domain was further described by specific constructs (*n* = 26) and subconstructs (*n* = 13) that can act as barriers or facilitators to implementation. Collectively, these 39 constructs and subconstructs are referred to as constructs throughout this paper.

**Figure 3:**
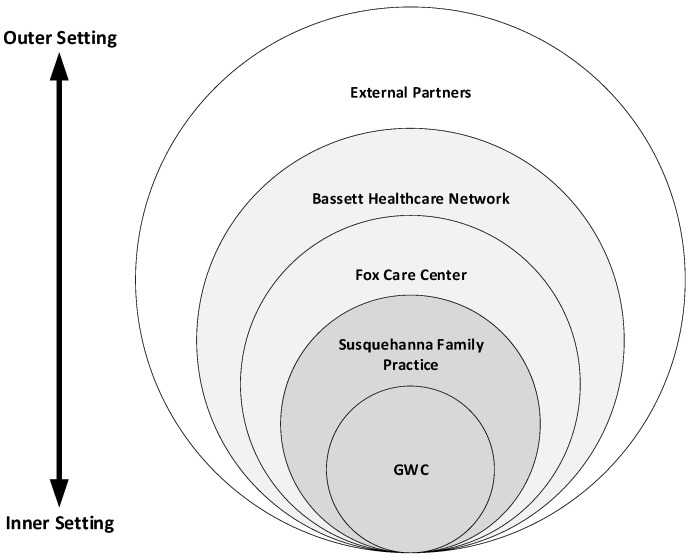
Depiction of the structure within which the GWC is embedded. For the purposes of this study, the GWC and Susquehanna Family Practice (dark grey) were considered the inner-most setting, with Fox Care Center and the Bassett Healthcare Network (light grey) displaying qualities of both the inner and outer settings, and partners external to Bassett Healthcare Network (white) in the outer-most setting.

The baseline survey included 76 primarily open-ended questions that included 28 CFIR constructs. Using the results of the baseline survey, the instrument was modified prior to follow-up. Constructs were removed from the survey instrument if they did not have any text coded to them during analysis of the baseline surveys. Some questions were modified to reflect that the COE had progressed further into the implementation phase. During the first survey, participants were confused about what was considered inner versus outer settings, partially due to a limited understanding of the CFIR and partially due to unclear distinctions between layers of the healthcare network as inner or outer settings (Figure 3). Therefore, questions were modified by naming specific parts of the healthcare network rather than asking directly about the inner and outer settings. Thus, these questions were often repeated in the follow-up survey for multiple parts of the healthcare network. The final version of the follow-up survey contained a total of 75 questions pertaining to 27 CFIR constructs. Appendix A includes a breakdown of baseline and follow-up survey questions by CFIR domain and construct in order to facilitate understanding of the CFIR as applied in this setting.

### 2.3. Survey Distribution

Baseline surveys were distributed to the core implementation team in April 2018 at the midpoint of the RWJF COE implementation project. These paper surveys were distributed via the second author, who is a member of the core implementation team and collaborator with the research team. Follow-up surveys were distributed to the core implementation team in October 2019 at the end of the grant cycle using Research Electronic Data Capture (REDCap), a web-based application for securely collecting and managing research data. Survey data were collected at two time points in order to understand changes in barriers and facilitators to implementation over 18 months.

### 2.4. Data Analysis

As the vast majority of survey questions required free text, a qualitative approach was used to analyze the data. Using NVIVO 12 [17], the first author open coded the free text survey responses. Individual codes were categorized into CFIR constructs. Though each survey question specifically related to one of the CFIR constructs, the open-ended nature of the questions allowed participants to elaborate on their responses. Open coding allowed the responses to be more accurately and specifically coded and categorized to the appropriate CFIR constructs. Additionally, this provided the opportunity to identify implementation factors not captured by the CFIR, though none were in this study. In addition to coding and categorizing text based on the CFIR, each coded segment of text was also rated using the −2 to +2 scale described by Damschroder et al. [13]. In this scale, positive numbers represent positive opinions or associations, negative numbers represent negative opinions or associations, and zero represents neutral opinions or associations. A value of +/−1 represents a general comment while +/−2 indicates that the participant described a specific example. Though limited interpretation of the data was necessary in order to summarize the data, the results presented reflected the manifest content of the survey responses, rather than latent meanings.

Given the challenges categorizing parts of the healthcare network as exclusively inner or outer setting, text relevant to each of these settings was coded based on the specific construct without consideration for the domain (inner or outer) within which those constructs fell. For example, though culture is a construct within the inner setting domain, it was also used to describe more outer setting layers such as the wider Bassett Healthcare Network.

## 3. Results

The results of this study are described in terms of each CFIR domain (intervention characteristics, process, characteristics of individuals, inner setting, and outer setting). These findings identified the constructs that act as both barriers and facilitators to implementation of this COE, as well as how these constructs changed from baseline to follow-up. Discussions of subconstructs were included under the main constructs under which they fell (i.e., engaging, implementation climate, and readiness for implementation).

### 3.1. Intervention Characteristics

Overall, there was little discussion of intervention characteristics in either the baseline or follow-up surveys. Four CFIR constructs did not have any text coded to them: design quality and packaging, intervention source, relative advantage, and trialability. Those constructs that were discussed saw a general trend of improved rating (from negative or neutral to positive rankings) over time, as well as an increase in specific positive examples at follow-up.

Evidence strength and quality: Over time, survey participants’ view of evidence supporting the COE changed in a positive way. At baseline, participants were largely aware of other COEs; however, they were unaware of the details or benefits of them and found it difficult to justify implementing their own COE. At follow-up, survey participants had more fully engaged in implementation of the COE and had seen firsthand evidence demonstrating the value of COEs, particularly in their own setting.

Complexity: Intervention complexity was a significant focus of participants during both the baseline and follow-up interviews. The tone in which participants discussed intervention complexity changed dramatically between the two surveys. At the time of the baseline survey, participants discussed the many moving parts of the COE (i.e., the five focus areas described in Figure 1, with numerous action items to achieve each part) as barriers to implementing the COE. At follow-up, these complexities were reframed as challenges, and participants indicated their success in overcoming most of them.

Adaptability: Though complex with some rigidity built into the strategic plan and six-pronged approach to implementing the COE, survey participants felt that the COE model could be adequately adapted to the current setting at baseline and demonstrated this adaptability in the examples provided during follow-up. Specific adaptations included those embracing the rural setting compared to urban settings that are traditionally home to COEs.

Cost: Concerns over the cost of maintaining the COE and the GWC within the larger healthcare network were discussed at both the baseline and follow-up. At baseline, several participants expressed concern about costs and indicated that the team needed more evidence to demonstrate financial sustainability. In the follow-up survey, this concern persisted; however, participants had begun thinking about funding options for continuing the GWC and COE, particularly in terms of competitive grants to establish and maintain relevant services.

### 3.2. Process

Similar to intervention characteristics, there was limited discussion related to process measures, particularly in the baseline surveys. Those discussions that did take place were more positive at follow-up than at the baseline.

Engaging: The CFIR construct of engaging can relate to a number of individuals. In this study, the primary focus of discussions related to engaging clinical and administrative champions; specifically, those who could serve as a bridge between the GWC and larger healthcare network. At baseline and follow-up, the discussions primarily focused on the fact that such champions were needed in order to make progress and establish sustainability within the network. Despite success in garnering support from many members of the healthcare network, including local administrators, there was a consistent concern over the difficulty of engaging key administrators in the network executive leadership team to actively support the COE and GWC. Both at baseline and follow-up, participants also recognized the importance of engaging members of the transgender population. Participants described their development of a community advisory board comprised of transgender individuals to address this gap.

Planning: Survey participants primarily discussed the planning construct in the baseline survey, which took place soon after the strategic plan for implementing the COE was developed and initiated. In these discussions, participants described the benefits of including transgender patients in the planning process as well as the importance of developing a cohesive plan for project implementation. At follow-up, this discussion transitioned to appreciation of the strategic plan as a valuable tool for implementation and evaluation of progress toward achieving stated goals.

Executing: Discussion of how the COE implementation would be executed did not occur at baseline. However, at follow-up, several participants discussed both challenges and facilitators of execution. Participants described challenges around involving nonclinical staff with the COE, particularly those who were not engaged in the process early on or who did not share similar values. Additionally, participants discussed the development of a marketing plan for the GWC, with conflicting feedback regarding how well this strategy was carried out.

Reflecting and Evaluating: The benefits of strategic planning were demonstrated further at follow-up when participants reflected back on their planned processes and goals. Participants described a process of repeatedly evaluating progress toward implementation of goals and revising the strategic plan based on challenges or new opportunities. Reflection and evaluation took place in the form of gathering patient, community, and healthcare network feedback through GWC advisory boards as well as team discussions regarding progress.

### 3.3. Characteristics of Individuals

Particularly when discussing the GWC team, participants reported positive views of constructs within the characteristics of individuals’ domain, which remained largely unchanged from baseline to follow-up. Much of this discussion took place during the baseline survey, with relatively little discussion of the various characteristics of individuals at follow-up.

Individual identification with the organization: Participants largely indicated that they themselves had beliefs that closely aligned with the mission of the GWC. Participants also indicated that some nonclinical or supervisory staff did not share similar values or passion for the work. Those individuals were reportedly more likely to hold personal biases about the gender-affirming care conducted at the GWC, creating challenges in implementation of the COE.

**Figure 4:**
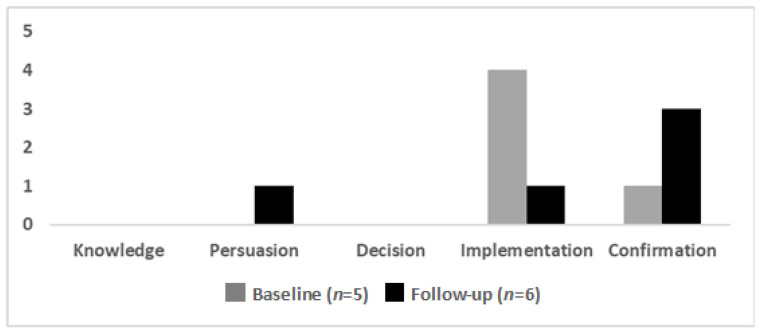
Stage of change at baseline and follow-up for participants to both surveys (*n* = 4). One individual responded to the baseline survey only, while two responded to the follow-up survey only.

Individual stage of change: At baseline, four of the five participants reported being in the implementation stage, with one reporting being in the confirmation stage (Figure 4). At follow-up, most participants (four of six) reported being in the confirmation stage, with one participant each in the implementation and persuasion stages (Figure 4).

Knowledge and beliefs about the intervention: Similar to individual identification with the organization, most participants indicated (at baseline and follow-up) positive opinions about the COE and implementation within the GWC. In rare instances, participants indicated that they were unsure of the overall success of the COE but still believed in the importance of the overall mission. There was also discussion about external individuals and the groups who were critical of the COE and concern that these opinions could damage team efforts.

Self-efficacy: Discussions of self-efficacy were notably limited. At baseline, participants indicated that they believed in their abilities to implement the COE.

Other personal attributes: Similar to other constructs captured in the characteristics of individuals’ domain, the codes categorized within the other personal attributes’ construct related primarily to individual’s intrinsic desires to succeed with the COE implementation. These discussions did not change significantly from baseline to follow-up.

### 3.4. Inner Setting

Throughout this study, constructs considered under the inner setting domain were among the most highly discussed. The relative positivity of the constructs was fairly static from baseline to follow-up.

Culture: In terms of this implementation study, organizational culture needed to be examined on multiple levels: from within the GWC, as well as within the outer layers of the healthcare network. As survey participants described at both baseline and follow-up, the organizational culture within each layer of the healthcare network was complex with differences between and within each layer. As the layers of the healthcare network interacted around COE implementation, these complexities and differences became more apparent. Overall, this became one of the most heavily discussed constructs among participants, with two primary components of culture discussed: the GWC culture (and resulting interactions) and culture surrounding the acceptance of trans-identified people.

One of the most direct survey questions related to culture asked participants, “to what extent (%) would you characterize your culture as” each of four types, with the end result totaling 100%. These types included:

1. Team: A friendly workplace where leaders act like mentors, facilitators, and team builders. There is value placed on long-term development and doing things together.
2. Hierarchical: A structured and formalized workplace where leaders act like coordinators, monitors, and organizers. There is value placed on incremental change and doing things right.
3. Entrepreneurial: A dynamic workplace with leaders that stimulate COE. There is value placed on breakthroughs and doing things first.
4. Rational: A competitive workplace with leaders like hard drivers, producers, or competitors. There is value placed on short-term performance and doing things fast.

**Figure 5:**
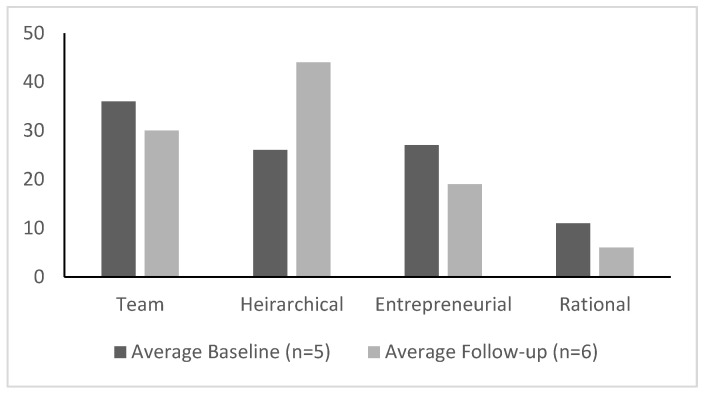
Average distribution of perceived team culture at baseline and follow-up.

Within the GWC itself, measures of the type of workplace culture most actively present changed over time, with the primary culture shifting from a mixture of team and entrepreneurial at the baseline to hierarchical at follow-up (Figure 5). In addition, at both baseline and follow-up, participants described relatively high amounts of conflict and negativity within the GWC but outside of the core implementation team. At baseline, there was a great deal of discussion about GWC and family practice staff feeling out of touch with the COE implementation and, therefore, without support. At follow-up, these concerns had improved.

In addition to these challenges, struggles related to support for the transgender population and the work of the GWC were present at both baseline and follow-up. At baseline, participants indicated that members of the GWC team were highly supportive of and provided affirming care to the transgender population. However, participants also indicated that outside of the GWC, individuals, including support staff in their own family practice, were sometimes less tolerant or supportive.

At follow-up, several participants indicated that as the GWC became better known and integrated into the larger healthcare network, there was a positive shift in network culture, with outside clinicians and nonclinical staff becoming more tolerant and affirming of the transgender population. Despite verbal support, a lack of follow-through with supportive actions often occurred, indicating that a full culture shift toward support of the GWC COE and transgender people had not yet been achieved.

Implementation climate: Much of the discussion on implementation climate focused on the inner-most implementation setting, the GWC itself. In this setting, participants generally were quite positive, describing an organizational incentive of improving patient care through the COE implementation. They described a positive learning climate with opportunities for education and the discussion and implementation of new ideas, as well as an appreciation for the strategic plan as a means of monitoring of goals and reviewing feedback.

Though most indicators of implementation climate were positive, there were mixed opinions of the compatibility of the COE with the existing environment at the time of the baseline survey. In addition, at both baseline and follow-up, participants described numerous competing priorities, particularly when it came to the responsibilities of the larger healthcare network. Finally, tension for change was shown to be variable in different parts of the healthcare network. The highest levels of tension for change were present in the immediate GWC; lower levels of tension for change existed in the more outer settings of the healthcare network.

Networks and communication: Communication issues, in particular, have been a challenge within the GWC over the course of the COE implementation. At both baseline and follow-up, participants indicated that communication within the GWC could be both tense and inconsistent. In terms of consistency, this related to both a lack of a standardized process for sharing new information, as well as challenges caused by the fact that several GWC mental health providers were not employed by the healthcare network and, therefore, unable to access patient records. At follow-up, participants expressed that communications about the GWC with other members of the larger healthcare network had improved over the course of the COE implementation.

Readiness for implementation: Due to the fact that the follow-up survey was conducted at the end of the grant cycle, much of the discussion related to readiness for implementation took place in the baseline survey. These constructs (leadership engagement, available resources, and access to knowledge and education) were described to a lesser extent in the follow-up survey.

Over the course of the study, participants indicated that network leadership engagement and support generally varied from person to person; however, at follow-up, participants indicated that there was overall more and better engagement by and support from leadership than at the baseline. At follow-up, several participants focused heavily on the resources that were lacking, specifically ancillary staff (mental health providers, administrative support, etc.), physical space, and time and energy of members of the GWC team. This lack of resources created barriers to smooth implementation of the COE. In addition, participants consistently described the educational opportunities that were available to them, noting, however, at baseline that individual’s ability to participate was somewhat limited due to time and funding constraints related to the consuming nature of project implementation.

Structural characteristics: From baseline to follow-up, responses did not change with regard to structural characteristics that could impact implementation. At both times, participants indicated that the relative newness of the GWC could be seen as a benefit to implementation, as there was ample opportunity to mold the GWC and COE to fit within the mission and vision of the larger healthcare network. While this was seen as a positive, COE implementation occurred during the restructuring of the healthcare network. Because this restructuring led to incorporation of the GWC into a new system, views were mixed with regard to the advantages and disadvantages of being included in a much larger network. This restructuring resulted in challenges adapting to a new organizational structure.

### 3.5. Outer Setting

Discussion of the outer setting constructs was limited in this analysis. Overall, the interactions between the GWC team, implementation of the COE, and the outer setting remained positive from the baseline through follow-up.

Cosmopolitanism: At both the baseline and follow-up surveys, participants discussed the benefits of collaborating with transgender healthcare providers outside of the healthcare network. Study participants indicated that making these connections through engagement in events and conferences was encouraged by GWC leadership.

External policy and incentives: Survey participants described the benefits of being the only organization in the region that provided interdisciplinary gender-affirming healthcare across the lifespan, with a commitment to the GWC’s six prongs for care (discussed previously). In addition, the implementation of the COE to improve these services was seen as positive compared to other regional services for transgender people.

Patient needs and resources: Throughout the study, survey participants consistently indicated that recognition of patient needs and the ability to meet those needs were a high priority for the GWC. At baseline, participants expressed concern that nonclinical GWC staff were less sensitive to the unique needs of transgender patients. This led to efforts to improve cultural competency through training and education. At follow-up, better patient transportation and health insurance were also identified as sources of gaps in care.

Peer pressure: At the baseline evaluations, participants described examples of peer pressure. This included the pressure to fit into the wider healthcare network, as well as the pressure to train other clinical departments within the healthcare network that also provide care for transgender patients. At follow-up, discussion around peer pressure focused on whether or not the GWC could continue to meet the healthcare network’s standards in terms of productivity and patient care metrics.

## 4. Discussion

This study highlights the implementation barriers and facilitators in the creation of a rural-based COE for transgender healthcare, which requires socio-cultural change in both the inner and outer settings of the health system. The multiple barriers to care and the stigma faced by transgender people require complex interventions, particularly in rural settings such as the one explored here. The results of this study are equally useful for GWC, aiming to improve implementation of the COE and sustain its efforts, as well as those outside of the GWC hoping to implement similar initiatives. These complexities can be seen in several individual constructs, as well as the interactions between them, as they have been described by participants in this study. Of particular interest in this study are the constructs within the inner and outer settings, which have created challenges to progress in assuring affirming care and fulfilling staffing needs, two arms of the COE strategy.

The constructs of organizational culture in the inner setting domain, engagement in the process domain, and individual stage of change and identification with the organization constructs in the characteristics of individuals’ domain were particularly important to implementation of the GWC’s COE. As previously described, while organizational culture is formally classified under the inner setting domain, this study considered the construct in terms of both the inner and outer settings. Similarly, engagement, which falls under the process domain, and the characteristics of individuals’ domain consider an array of inner and outer setting individuals. Prior studies have demonstrated that characteristics of individuals constructs are closely related to culture and engagement and together can influence implementation [18,19].

Comparing the baseline and follow-up surveys demonstrated a more positive outlook expressed during follow-up, despite ongoing challenges within the inner setting, including communication problems, lack of financial and physical space resources, tension over fitting into the network, and relative priority of the GWC compared to other initiatives within the healthcare network. Overall, the culture of the inner-most setting (GWC) was reported to be highly trans-affirming, while the outer settings, i.e., other parts of the healthcare network, were less affirming and less consistent in accepting and supporting people with diverse gender identities. It is likely that the differences in organizational cultures are closely connected to the characteristics of the individuals working in each setting. The relationship between individuals and their culture has the potential to affect constructs such as individual identification with the organization, individual stage of change, knowledge and beliefs about the intervention, and self-efficacy in both positive and negative ways.

Less trans-affirming attitudes and actions within healthcare networks is usually attributed to a lack of education and training regarding transgender people, their healthcare disparities, and their specific healthcare needs. Stigma and transphobia are well described and pervasive throughout healthcare cultures across the United States [4]. Trainings in cultural competency and responsiveness are effective in increasing staff awareness and understanding of the transgender community and lead to decreased discrimination [20,21]. Though it was outside of the scope of this study to assess the other subcultures within the network, prior studies indicate that network training and education regarding cultural competency will be essential to wider network culture change.

The GWC’s individual and organizational cultural influences, and those of the larger healthcare network, as well as those outside of the network impact how individuals engage in COE activities [22]. While study participants reported active support from many network administrators, staff, and clinicians, they also described a lack of meaningful engagement with some individuals in top leadership positions, including those most critical to champion efforts. Thus, at both baseline and follow-up, study participants described numerous competing priorities, particularly when it came to responsibilities set forth by the larger healthcare network. While it is possible that this lack of engagement stems from individual beliefs or organizational values regarding transgender healthcare, we did not assess the leadership’s level of engagement and, thus, we cannot comment further based on the results of this study. However, the complexity of engaging certain network-wide, shared services in all aspects of the network at all times is significant. In many cases, it may not be feasible for various specialty or primary care services to be as engaged in implementation as the GWC team would like. This is particularly true in a time of healthcare restructuring that ran parallel to the COE implementation.

Despite remaining challenges, the GWC team created a center with an excellent reputation and increased regional visibility. The GWC was successful in developing its infrastructure, expanding clinicians’ knowledge and skills in the area of transgender care, and advancing evidence-based practices–the remaining three arms of the COE strategy. Increasing numbers of patients sought care, often from areas outside the usual network catchment area. Despite increased patient volume, participants described financial successes and vulnerabilities. Several survey participants noted that the network was focused on financial viability whereas the GWC was focused on providing highly specialized care to a vulnerable minority, a setup for organizational conflict. Though these conflicts existed at both baseline and follow-up, there were improvements over the course of the COE implementation, likely due to the increased visibility of the GWC and movement toward the normalization of gender-affirming care. To further address concerns over the value of the GWC within the healthcare network, the GWC team developed a business plan to demonstrate its financial and cultural worth to the network. This included documentation of the GWC’s financial growth and sustainability, importance as a research and training institution, and plans to obtain additional grant funding for projects unrelated to direct patient care.

The GWC team developed a network of individuals within and outside the network who served as allies and change agents for project implementation, including administrative champions, patients, colleagues, and representative from other LGBT organizations across the state. These social networks allowed the GWC team to engage with people and programs with a similar interest in reducing healthcare disparities for at-risk populations.

Additional discussions focused on the complexity of the intervention, an issue related to self-efficacy of the GWC team, its knowledge of COEs, and beliefs about how implementation of a COE would affect its work. At baseline, complexity of the COE and implementation process was discussed as a barrier to implementation. At follow-up, complexity was seen as a challenge rather than a barrier. This suggests that firsthand experience in COE implementation led to a deeper understanding of the process, more positive knowledge and beliefs about providing gender-affirming care, and improved self-efficacy in the provision of care. The development and regular review of the COE strategic planning document helped to ease perceived complexities, creating a roadmap for implementation.

The constructs within the inner and outer setting domains were discussed in survey responses far more frequently, and with far more detail, than most others. This indicates the important influence, either positive or negative, that they had on implementing the COE compared to other constructs. Other constructs, such as networks and communications, presented challenges, while yet others (e.g., cosmopolitanism) provided additional support for implementation. Participant feedback did not suggest that any one of these factors was solely responsible for the overall success of the COE implementation.

## 5. Strengths and Limitations

The primary limitation of this study is the small number of participants. While this is recognized as an important limitation, it is also relevant that all individuals who were part of the core implementation team were invited to participate, with one at baseline and none at follow-up declining participation. As this study collected qualitative data, the data were richer and more nuanced than that obtainable through quantitative evaluation methods. An additional limitation is that the first survey was conducted using a handwritten paper form, while the second survey was conducted using an electronic survey. Because of the makeup of the study population (individuals invested in implementation of the COE), it is unlikely that the study response rate was impacted by the survey method. However, it is possible that the handwritten survey resulted in less detail than the electronic survey. Though true, participants were presented with a summary of results after both the baseline and follow-up surveys were completed and indicated that their opinions were adequately captured at both times.

Several strategies were employed to increase the trustworthiness of the data and analysis, as suggested by Graneheim et al. [23]. First, the primary author is not employed by the GWC and, thus, provides an outside perspective, whereas the remaining authors are either employed within the GWC or have worked closely with the GWC on other initiatives. Second, the primary author presented both baseline and follow-up results to the GWC staff in order to confirm results and gather additional feedback. This process demonstrated that the analysis was on par with participants’ experiences. These methods of triangulation help to increase both credibility and confirmability of the data. In addition, because the data were collected at two time points, 18 months apart, the results can be considered dependable, in that key concepts were assessed and confirmed over time.

## 6. Conclusions

This study highlights the successes and challenges faced by a clinical team as it implemented a rural-based COE in transgender health. As described in Figure 1, five COE implementation and strategic plans focused on five areas. The results of this study highlight success in three of these areas (developing infrastructure and organizational capacity; expanding awareness, knowledge, and skills; and advancing evidence-based care). The remaining two areas (fulfilling staffing needs and ensuring affirming care) also saw some successes but were constrained by limitations of the inner and outer setting. The challenges described in this study include, and underscore the importance of, providing culturally appropriate care and environments to underserved populations, such as transgender people. The results of this case study will allow others trying to implement a COE to better understand the barriers and facilitators to implementation of a COE and, in so doing, assist them in anticipating and addressing these in their implementation process.

Additionally, the results of the study provide some context and guidance for others aiming to implement similar initiatives. A clear assessment of the organizational culture in the inner setting and maximal engagement of leaders in both inner and outer settings will facilitate effective implementation of the intervention. This study shows the usefulness of the CFIR process in analyzing a complex intervention (CEO creation) in addressing a wicked problem (lack of transgender care). The domains and constructs provide a vocabulary and syntax that help to describe and analyze this complex intervention. One limitation of the CFIR process may be ambiguity in where to draw the boundary between inner and outer settings.

## Abbreviations

CFIR:	Consolidated Framework for Implementation Research
COE:	Center of Excellence
GWC:	Gender Wellness Center

## Appendix A

**Table A1:** Survey Questions by CFIR construct.

Constructs	Questions ^a,b^
**Intervention Source**	N/A
**Evidence Strength and Quality**	What kind of information or evidence are you aware of that shows whether or not the COE will work [worked] in your setting? What kind of supporting evidence or proof is needed about the effectiveness of the COE to get staff on board? *Baseline only* In a healthcare setting, influential stakeholder may include influential and well-respected clinicians. What do influential stakeholders think of the COE? What do administrative or other leaders think of the COE?
**Relative Advantage**	N/A
**Adaptability**	What kinds of changes or alterations for you think you will need to make the COE so it will work effectively in your setting? Who will decide (or what is the process for deciding) whether changes are needed to the COE so that it works will in your setting? Are there components that should not be altered? Which ones?
**Trialability**	N/A
**Complexity**	How complicated is the COE?
**Design Quality and Packaging**	N/A
**Cost**	N/A
**Patient Needs and Resources**	To what extent is staff aware of the needs and preferences of the individuals being served by the GWC? To what extent were the needs and preferences of the individuals served by the GWC considered when deciding to implement [while implementing] the COE? How well do you think the COE will meet [is meeting] the needs of the individuals served by the COE? How do you think the individuals served by the GWC will respond to the COE? [How did individuals respond?] What barriers will the individuals served by the GWC face to participating in the COE? [What barriers did individuals face?] Have you ever elicited information from participants regarding their experiences with the COE? Have you heard stories about the experiences of participants with the COE?
**Cosmopolitanism**	To what extent do you network with colleagues or people in similar professions/positions outside your setting? What kind of information exchange do you have with others outside your setting, either related to the COE or more generally about your profession? *Baseline only.*
**Peer Pressure**	To what extent does implementing the COE provide an advantage for Fox Care Center compared to other organizations in your area? *Follow-up only* Can you tell me what you know about any other organizations that have implemented the COE or other transgender health care services? To what extent are other units within the Bassett Healthcare Network implementing transgender health care services? To what extent would implementing the COE provide an advantage for the Bassett Healthcare Network compared to other healthcare providers in your area?
**External Policies and Incentives**	What kind of local, state, or national performance measures, policies, regulations, or guidelines influenced the decision to implemented the COE? *Baseline only.* What kind of financial or other incentives influence the decision to implement the COE? *Baseline only.*
**Structural Characteristics**	How will [did] the infrastructure of the GWC (social architecture, age, maturity, size, or physical layout) affect the implementation of the COE? What kinds of infrastructure changes were or will be needed to accommodate the COE? What changes in the design and packaging of the GWC and/or the COE affect implementation? *Follow-up only.*
**Networks and Communications**	Can you describe your working relationships with your colleagues? Do you meet (formally or informally) with a team of people? Can you describe your working relationship with leaders? Can you describe you working relationship with influential stakeholders? Are meetings, such as staff meetings, held regularly? How do you typically find out about new information, such as new initiatives, accomplishments, issues, new staff, or staff departures? When you need to get something done or to solve a problem, who are your “go-to” people?
**Culture**	How would you describe the culture of the Bassett Healthcare Network? How would you describe the culture for Fox Care Center? *Follow-up only.* How would you describe the culture for the GWC? *Follow-up only.* Do you feel like the culture of the GWC is different from the overall organization? In what ways? How do you think the Bassett Healthcare Network’s culture (general beliefs, values, assumptions that people embrace) will affect [affected] the implementation of the COE? To what extent are new ideas embraced and used to make improvements in the Bassett Healthcare Network? Some people characterize culture in terms of four general types. To what extent (%) would you characterize your culture as: team (clan) culture; hierarchical (hierarchy) culture; entrepreneurial (adhocracy) culture; rational (market) culture?
***Implementation Climate***	
**Tension for Change**	In there a strong need for this COE? How essential is this COE to meet the needs of the individuals served by the [Fox Care Center or] Bassett Healthcare Network or other organizational goals and objectives? How do people feel about current programs/practices/processes that are available related to the COE?
**Compatibility**	How well does the COE fie with your values and norms? How well does the COE fit with the values and norms within the Bassett Healthcare Network? How well does the COE fit with existing work processes and practices in your setting? Can you describe how the COE will be integrated into current processes? *Baseline only.* How well did the COE integrate into current processes at the GWC or Susquehanna Family Practice? *Follow-up only.* Will the COE replace or compliment a current program or process? *Baseline only.*
**Relative Priority**	What kinds of high priority initiatives or activities are already happening in your setting? Describe activities or initiatives that (appear to) have highest priority for you (for the organization). *Baseline only.* To what extent might the implementation take a backseat to other high-priority initiatives going on now? How will you juggle competing priorities in your own work? How will your colleagues juggle these priorities? What is the general level of receptivity in your organization [at Fox Care Center, at Bassett Healthcare Network] to implementing the COE?
**Organizational Incentives and Rewards**	What kinds of incentives are there to help ensure that the implementation of the COE is successful? Have you, the GWC, Fox Care Center or Bassett Healthcare Network set goals related to the implementation of the COE? If so, what were these goals? *Follow-up only.* How does [did] implementation of the COE align with other organizational (network level) goals?
**Goals and Feedback**	N/A
**Learning Climate**	N/A
***Readiness for Implementation***	
**Leadership Engagement**	What level of endorsement or support have you seen or heard from leaders? What level of involvement has leadership at your organization had so far with the COE? What kind of support or actions can you expect from leaders in your organization to help make implementation successful?
**Available Resources**	Do you expect to have [Did you have] sufficient resources to implement and administer the COE? Do you expect to have sufficient resources to administer the COE in the future? *Follow-up only.* How do you expect to procure necessary resources?
**Access to Knowledge and Information**	N/A
**Knowledge and Beliefs about the Intervention**	Do you think the COE will be [has been] effective in your setting? At what stage of implementation is the COE at in your organization?
**Self-efficacy**	How confident are you that you will be able to successfully [continue] implement[ing] the COE? How confident are you that you will be able to use the COE? *Baseline only.* How confident do you think your colleagues feel about implementing [continuing] the COE?
**Individual Stage of Change**	How prepared are you to use [continue] the COE? (Knowledge, Persuasion, Decision, Implementation, or Confirmation stage).
**Individual Identification with Organization**	N/A
**Other Personal Attributes**	N/A
**Planning**	What role has your strategic plan for implementation played during implementation? Was the COE implemented according to the strategic plan? *Follow-up only.* Looking back, how satisfied are you with your ability to implement the COE strategic plan? *Follow-up only.*
***Engaging***	
**Opinion Leaders**	Who are the key influential individuals to get on board [continue] with this implementation? What are influential individuals saying about the COE?
**Formally Appointed InternalImplementation Leaders**	N/A
**Champions**	Other than the formal implementation leader, are there people in your organization who are likely to champion (go above and beyond what might be expected) the COE? What kinds of behaviors or actions do you think this individual/champion will exhibit? *Baseline only.*
**External Change Agents**	Will someone (or a team) outside your organization be helping you with implementing [continuing] the COE?
**Executing**	Is the COE being implemented according to the strategic plan? *Baseline only.* What is [was] your communication or education strategy (not including training) for getting the word out about the COE?) How will [did] you or your colleagues communicate to the individuals that are served by the GWC about the COE?
**Reflecting and Evaluating**	What kind of information do you plan to collect as you implement the COE? *Baseline only.* To what extent are organizational goals monitored for progress? *Follow-up only.* Will [Did] you receive feedback reports about the implementation of the COE? How will [did] you assess progress toward implementation of COE goals? Will feedback be [Was feedback] elicited from staff? From individuals served by the GWC? To what extent has you organization/unit set goals for implementing the COE? *Baseline only.* To what extent has the GWC met goals for implementing the COE? *Follow-up only.*

^a^ Unless otherwise noted, questions were repeated at baseline and follow-up. In instances in which the phrasing was changed, the phrasing at follow-up is shown in square brackets. ^b^ Only primary questions are presented in this table. Participants were also asked to explain their responses using question prompts. For example, for the Networks and Communications domain question, “Are meetings, such as staff meetings, held regularly?” prompts included: “Do you typically attend? Who typically attends? What proportion of staff typically attend? How often are the meetings held? What is a typical agenda? How helpful are these meetings?”.
